# Simultaneous Expression of Abiotic Stress Responsive Transcription Factors, *AtDREB2A, AtHB7* and *AtABF3* Improves Salinity and Drought Tolerance in Peanut (*Arachis hypogaea* L.)

**DOI:** 10.1371/journal.pone.0111152

**Published:** 2014-12-04

**Authors:** Vittal Pruthvi, Rama Narasimhan, Karaba N. Nataraja

**Affiliations:** Department of Crop Physiology, University of Agricultural Sciences, Bangalore, Karnataka, India; University of Antwerp, Belgium

## Abstract

Drought, salinity and extreme temperatures are the most common abiotic stresses, adversely affecting plant growth and productivity. Exposure of plants to stress activates stress signalling pathways that induce biochemical and physiological changes essential for stress acclimation. Stress tolerance is governed by multiple traits, and importance of a few traits in imparting tolerance has been demonstrated. Under drought, traits linked to water mining and water conservation, water use efficiency and cellular tolerance (CT) to desiccation are considered to be relevant. In this study, an attempt has been made to improve CT in drought hardy crop, peanut (*Arachis hypogaea* L., *cv.* TMV2) by co-expressing stress-responsive transcription factors (TFs), *AtDREB2A, AtHB7* and *AtABF3*, associated with downstream gene expression. Transgenic plants simultaneously expressing these TFs showed increased tolerance to drought, salinity and oxidative stresses compared to wild type, with an increase in total plant biomass. The transgenic plants exhibited improved membrane and chlorophyll stability due to enhanced reactive oxygen species scavenging and osmotic adjustment by proline synthesis under stress. The improvement in stress tolerance in transgenic lines were associated with induced expression of various CT related genes like *AhGlutaredoxin*, *AhAldehyde reductase*, *AhSerine threonine kinase* like protein, *AhRbx1*, *AhProline amino peptidase*, *AhHSP70*, *AhDIP* and *AhLea4*. Taken together the results indicate that co-expression of stress responsive TFs can activate multiple CT pathways, and this strategy can be employed to improve abiotic stress tolerance in crop plants.

## Introduction

Peanut, (*Arachis hypogaea* L.) an important oilseed crop, is a major source of edible oil and third most important source of vegetable protein, besides serving as a dietary source of vitamin E and phytosterols (FAO, 2010). The crop is cultivated in semi-arid regions in an estimated area of 23.95 million ha worldwide. Asia is the major peanut growing region in the world, accounting for 64% of the global production (FAOSTAT, 2011). Although peanut is considered as a dry land crop with multiple stress adaptation traits, drought in conjunction with high temperature stress is one of the important constraints for its productivity [1].

Drought tolerance in plants is a complex process governed by multiple pathways and genes [2]. Conventional breeding approach using one or two traits has not yielded satisfactory result due to lack of key genes underlying the QTLs [3]. In addition, the slow progress in drought resistance breeding is also due to limited characterization of drought tolerance traits [4]. Although molecular marker assisted breeding has been attempted in peanut, due to low level of polymorphism in cultivated varieties the results are not encouraging [4]. To overcome a certain limitation of classical breeding approaches, genetic engineering through transgenic approach has been attempted for targeted improvement of crop towards stress tolerance [3]. There are reports on improvement of plants using transgenic approaches in different field crops such as rice [5]–[7], maize [8], soybean [9], [10] and potato [11]. However, such attempts in peanut are limited [12], [13].

To improve water use efficiency (WUE, the most important drought tolerant trait) of peanut under drought condition, an attempt had been made to express transcription factor (TF), *AtDREB1A* in peanut [12]. Under water deficit condition, one of the transgenic lines showed 40% higher transpiration efficiency suggesting the importance of targeted genetic manipulation in peanut using single gene. Asif *et al* [13] demonstrated the usefulness of transgenic approach in improving drought and salt tolerance using vascular type sodium antiporter gene, *AtNHX1* cloned from *Arabidopsis*. *AtNHX1* expressing transgenic plants showed better performance under NaCl (200 mM) stress and recovery was faster in transgenic lines after drought alleviation. These studies indicate that transgenic approaches can be employed in peanut for crop improvement towards drought tolerance.

Although transgenic approach for abiotic stress tolerance through manipulation of single gene has been successful, alteration of complex interactive pathways and quantitative traits requires simultaneous expression of different upstream genes [14]. The coordinated expression of different genes can assist in the regulation of many stress responsive genes associated with specific resistance pathways. Among the different traits linked to drought tolerance, cellular tolerance (CT) has been suggested to be an important trait [15], [16]. Several transgenic approaches which have manipulated the CT through the over expression of gene involved in biosynthesis of osmolytes [17], scavenging of reactive oxygen species (ROS) [18], maintenance of transcriptional machineries and cell membrane stability [19], resulted in significant improvement in drought acclimation in different plants.

The TFs are considered as upstream regulatory proteins, which play a major role in cellular metabolism and abiotic stress response. Different TFs have been overexpressed in model systems and crop plants to improve stress tolerance [20], [21]. Many downstream stress genes have multiple stress responsive TF binding sites [22] indicating a complex network of pathways associated with stress acclimation in plants. Therefore, it would be beneficial to co-express candidate TFs for imparting better drought tolerance. From this context, in the present investigation, an attempt was made to co-express three different validated drought responsive TFs, namely *AtDREB2A, AtHB7* and *AtABF3* in peanut. Overexpression of *AtDREB2A* improved drought tolerance in *Arabidopsis* [23]. Independent expression of *AtABF3* and *AtHB7* resulted in improved stress tolerance in rice [7] and *Arabidopsis* [24], respectively. In this study, these three genes were constitutively co-expressed in peanut by developing stable transgenic plants using *in-vitro* regeneration protocol. This is one of the initial studies in peanut involving stacking of three different TFs to improve drought tolerance.

## Materials and Methods

### Vector construction

The multigene cloning strategy (Gateway Technology, Invitrogen, USA) was followed to develop binary vector. The full length *Arabidopsis* cDNA clones of *AtDREB2A, AtHB7* and *AtABF3* were procured from RIKEN Genomic Sciences Centre (GSC), Plant Functional Genomics Research Group (PFG), Japan. All the three genes, *AtDREB2A* (AT5G05410), *AtHB7* (At2G46680) and *AtABF3* (AT4G34000) were sub cloned to pRT100 vectors under CaMV35S promoter and polyA terminator at *Apa1* and *Nco1*, *Kpn1* and *Nco1,* and *Kpn1* and *BamH1* sites, respectively. The CaMV35S promoter and polyA terminator specific primers with *attB* recombinant sites (Table S1 in File S1) were used to amplify the full cassette from pRT100 vector by PCR and the product was cloned into specific pDONR vectors (as per manufacture’s protocol, Invitrogen, USA) by BP clonase driven recombination (BP) reaction. LR reaction was performed using the recombinant entry clones, *attL1*P_CaMV35S_ *::AtDREB2A-*T_PolyA_ *-attL4*, *attL4r-*P_CaMV35S_ *::AtHB7-*T_PolyA_ *-attL3r* and *attL3-* P_CaMV35S_ *:: AtABF3-*T_PolyA_ *-attL2* with destination (plant expression) vector *pKM12GW* [25], in the presence of LR clonase enzyme at 25°C overnight. The resulting recombinant vector (Figure S1a in File S1) was used to transform *Agrobacterium tumefaceins* (*LBA4404*) by electroporation (Eppendorf AG, Germany) [26].

### Plant material and transformation

Seeds of peanut (*Arachis hypogaea* L., *cv*. TMV2) were obtained from the National Seed Project (NSP), University of Agricultural Sciences, GKVK, Bengaluru, Karnataka, India. Seeds were surface sterilized with fungicide (0.1% w/v, carbendazim, BASF, Germany) for 1 h and further sterilized with 0.1% (w/v) mercuric chloride for 4–5 min and germinated on filter paper bridges containing sterile distilled water. Cotyledonary nodes were excised aseptically from 5–6 day-old seedlings and inoculated on to Murashige and Skoog’s (MS) medium containing 3% (w/v) sucrose. All the cultures were maintained at a temperature of 25±2°C under a 16/8-h (light/dark) photoperiod provided by cool white, fluorescent lamps. For plant transformation, *Agrobacterium* (strain *LB4404*) was grown at 28°C in AB minimal medium supplemented with kanamycin (50 mg/L) and used to infect explants for 4–5 min at room temperature. Infected explants were subjected for shoot initiation on MS media (shoot initiation media, SIM) containing BAP (3 mg/L), NAA (1 mg/L) and kanamycin (125 µg/mL). Once appreciable growth was seen, the explants were transferred on shoot proliferation media (SPM) containing BAP (3 mg/L), NAA (1 mg/L) and kanamycin (125 µg/mL). The shoots developed were transferred to shoot elongation media (SEM) containing GA (1 mg/L) and kanamycin (100 µg/mL) to induce shoot elongation. Putative transgenic plants were then transferred to root induction media (RIM) containing IBA (0.5 mg/L) and kanamycin (75 µg/mL). The rooted plantlets were transplanted into pots containing soilrite and covered with plastic bags to prevent dehydration, and subsequently allowed for hardening under controlled environmental conditions. After one week, the plants were transplanted to pots containing potting mixture and allowed to grow to maturity in the transgenic containment facility.

For selection and evaluation of transgenic lines, germinated seeds were soaked in kanamycin (400 ppm) for 5 h and subsequently transferred to sand medium supplemented with nutrient solution, and allowed to grow for 15 days. The plants with good root growth were selected and progressed to next generation.

### Molecular analysis of transgenic plants

Genomic DNA was isolated by cetyl trimethyl ammonium bromide (CTAB) method using young leaf tissue of wild type and transgenic peanut plants [27]. The transgenic lines were reconfirmed by PCR using marker gene, neomycin phosphotransferase (*nptII*) specific primers, and other transgene specific primers (Table S1 in File S1). The amplified products were sequenced to confirm the identity (Figure S2 in File S1).

### Total RNA isolation, qRT-PCR and RT-PCR analysis

Total RNA was isolated from leaves by following phenol chloroform method [28]. Total RNA was reverse transcribed to generate cDNA by using Revert Aid Reverse Transcriptase (MMLV-RT; MBI Fermentas, Hanover, MD, USA) using oligo (dT) primers (Table S1 in File S1) following manufacturer’s instructions. Real-time PCR was performed in the presence of SYBR-green fluorescence dye (DyNAmo SYBR-Green qPCR Kit FiNNZYMES, Finland) using equal amount of cDNA. The critical threshold cycle (Ct) values were normalized using Ct obtained for elongation factor-A (*ELF-A*) in respective samples and relative expression was calculated [29].

The downstream target genes of *AtDREB2A*, *AtHB7* and *AtABF3* were studied by RT-PCR analysis. The target gene sequences were obtained from peanut ESTs (http://www.ncbi.nlm.nih.gov) for designing primers (Table S1 in File S1).

### Stress imposition at seedling stage

Mature seeds of uniform size were soaked and allowed to grow on petri plates with NaCl (200 mM) and mannitol (200 mM) for one week. At the end of the stress period, root length (cm) and lateral root numbers were recorded. To study the recovery growth, stress exposed seedlings were allowed for recovery on sand medium supplemented with nutrients for 15 days. The root length (cm) and lateral root numbers were recorded 15 days after recovery.

#### Leaf disc assay

The trifoliate leaves of T_2_ generation plants of same age were taken from both wild type and transgenic plants. The leaf discs collected were floated in methyl viologen (5 µM) and ethrel (1200 ppm) (Sigma-Aldrich, USA) overnight under dark condition. Further, the tissue was exposed to high light (1200 µmol.m^−2^.s^−1^) for 1 h. Leaf tissue bleaching and lipid peroxidation was assessed by quantifying total chlorophyll [30] and malondialdehyde (MDA) [31], respectively. Similarly, the cell viability was assessed by TTC test [32].

#### Salinity and drought imposition at whole plant level

Salinity stress was imposed to three week old plants by irrigating with Hoagland’s solution containing NaCl (150 mM) for 4 days and subsequently treated with sub lethal dose of NaCl (250 mM) for 10 days and scored for chlorosis and tip burning. Total dry matter (g, DW: dry weight), total chlorophyll (mg.gDW^−1^), cell membrane stability (%), MDA (µM.gDW^−1^) and superoxide dismutase (SOD) activity were recorded at the end of the stress period.

Drought stress was imposed to 20 days old plants by withholding water until the required soil field capacity (FC, %) was reached and the required level of FC was maintained by gravimetric approach [6], [33]. The plants were maintained at maximum temperature of 28°C with light intensity of 1,000–1,200 µmol.m^−2^.s^−1^. Control plants were maintained at 100% (FC), while drought stressed plants were exposed to 60–70 and 20–30% FC for two weeks. Subsequently, plants were irrigated to 100% FC and phenotype after recovery was recorded.

### Measurement of physiological parameters

#### Relative water content (RWC)

The RWC in leaf tissue was quantified according to Barrs and Weatherly [34]. After recording the fresh weight, the leaf discs were floated on deionized water for 5 h at 28°C to determine the turgid weight. Dry weight was determined after oven drying to a constant weight.

#### Measurement of photosynthesis

Photosynthetic parameters were recorded using the portable photosynthetic system (LICOR 6400, USA) on healthy leaves of 45 days old plants. Net photosynthesis (A, µmol.m^−2^.s^−1^) and stomatal conductance (gs, mmol.m^−2^.s^−1^) were recorded at an ambient CO2 concentration of 360 µmol.mol^−1^ and PPFD of 1200 µmol.m^−2^.s^–1^ using LICOR light source and chamber temperature of 28°C±0.5 [35]. The *in-vitro* PSII activity (Φ_PSII_) was also analysed [36].

### Estimation of biochemical parameters

#### Cell membrane stability (CMS)

Leaf discs were incubated in deionised water for 8 h at 25°C. Extract of electrolyte that leaked into bathing medium was recorded (T1) using conductivity bridge. Subsequently, the leaf segments were boiled for 30 min and allowed to cool down and final reading was recorded (T2). Similarly, leakage was also measured from non-stressed plants. The CMS was calculated using the formula, CMS (%) = [1–(T1/T2)]/[1–(C1/C2)]×100 where, C1 and C2 are the initial and final readings, respectively recorded in non-stressed tissue [37].

#### Estimation of leaf total chlorophyll

Leaf chlorophyll content was quantified by taking 100 mg fresh leaf tissue. Total chlorophyll was extracted in a 1∶1 mixture of dimethyl sulfoxide (DMSO) and acetone (80%, v/v) solution overnight under dark. Optical density (OD) was recorded at 663 and 645 nm using spectrophotometer (SpectroMax plus, Molecular Devices, USA). Total chlorophyll was estimated and expressed as mg.gDW^−1^ [30].

#### Estimation of malondialdehyde (MDA)

About 0.5–1.0 g of tissue was homogenized in 5 ml of 5% (w/v) trichloroacetic acid, and used for the analysis. Absorbance of the reaction mixture was measured at 532 nm using spectrophotometer, (SpectroMax plus, Molecular Devices, USA) and corrected for nonspecific turbidity by subtracting the A_600_. The MDA equivalents were calculated by the extinction coefficient of 155 M^−1^.cm^−1^ [31].

#### Estimation of cell viability by 2, 3, 5-triphenyl tetrazolium chloride (TTC) assay

The TTC assay was performed to measure the cell viability. Leaf discs were incubated in TTC solution at room temperature for 5 h under shaking and the bound TTC was extracted and absorbance was measured at 485 nm using UV-visible spectrophotometer (SpectroMax plus, Molecular Devices, USA) [32].

#### Nitro blue tetrazolium (NBT) assay to study SOD activity

Leaf samples were used to estimate NBT reduction according to Beyer et al [38]. Leaf samples (100 mg) were homogenised in potassium phosphate buffer (0.5 M, pH 7.2) and exposed to the reaction in a buffer containing methionine (300 mg/10 mL), NBT (14.1 mg/10 mL), tritonx100 (1%) and riboflavin (4.4 mg/10 mL). The samples were incubated in light (500 µmol.m^−2^.s^−1^) until blue colour appears. The colour intensity was recorded at 560 nm using a spectrophotometer (SpectroMax plus, Molecular Devices, USA).

#### Proline estimation

Proline content was estimated [39] based on proline’s reaction with ninhydrin. Accordingly, a 1∶1∶1 (v/v/v) solution of plant extract, acid–ninhydrin and glacial acetic acid was incubated at 100°C for 1 h. The reaction was arrested on an ice bath and the chromophore was extracted with 4 ml toluene and its absorbance at 520 nm wavelength was determined in a spectrophotometer (SpectroMax plus, Molecular Devices, USA).

## Results

### *In-vitro* regeneration and transformation of peanut

Cotyledonary nodes excised from six day old seedlings were used to generate transgenic peanut plants by *Agrobacterium* mediated transformation. Over 400 putative transgenic plants were developed and screened *in-vitro* and healthy rooted plantlets were successfully grown up to maturity. Although regeneration protocol yielded sufficient number of shoots after transformation, only 30% of shoots produced healthy roots in RIM as there was inhibition of root induction in medium containing kanamycin, as reported earlier [40]. The different stages of *in-vitro* regeneration transformation protocol are presented in the Figure S1 (b–g) in File S1. More than 75 independent T_1_ transgenic lines were selected, grown in transgenic containment facility and subjected for molecular analysis. Two best performing independent transgenic lines (named as L1 and L7) were selected for the study based on their enhanced tolerance under drought in the previous generation.

**Figure 1:**
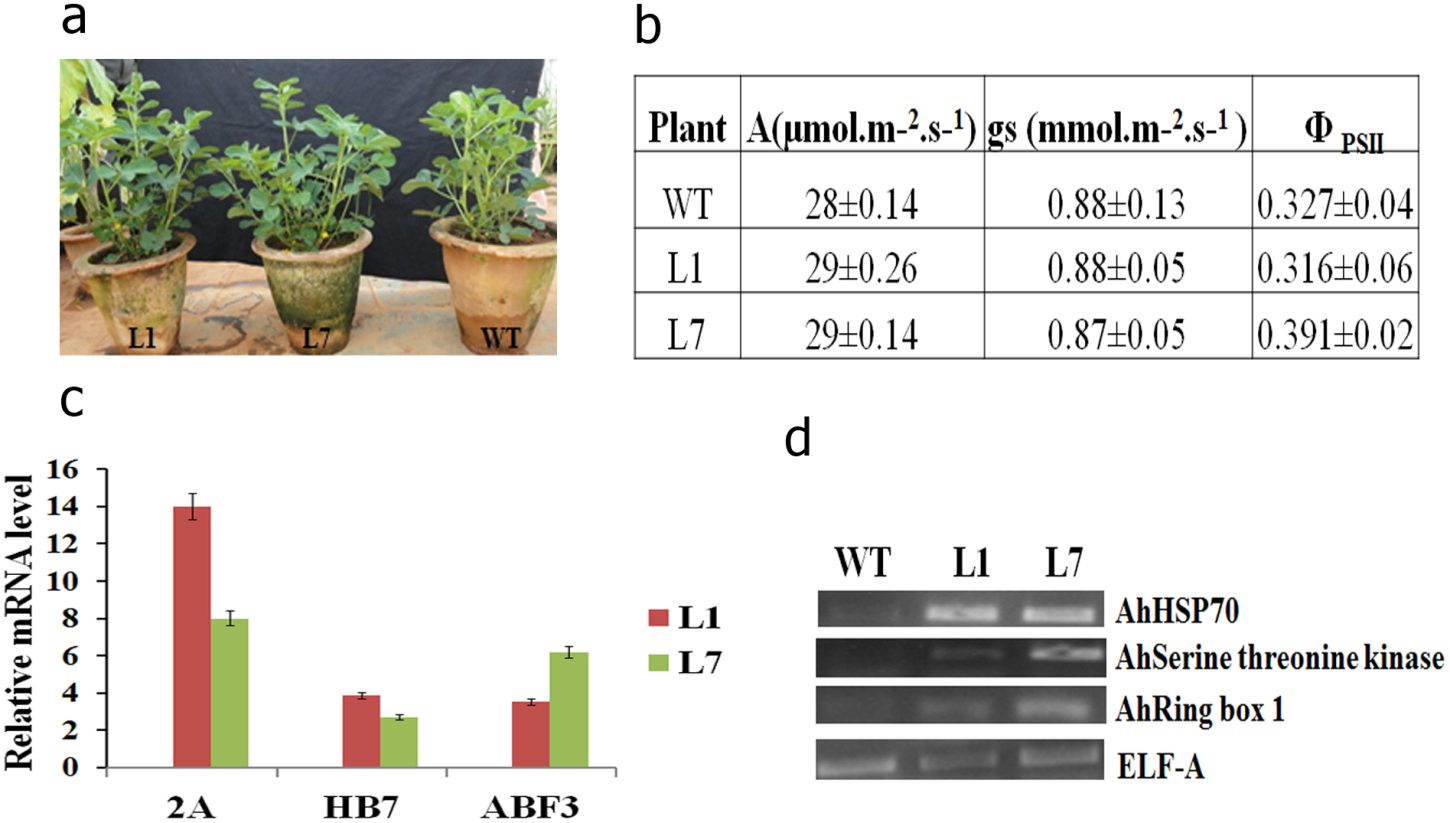
Characterization of peanut transgenic plants co-expressing *AtDREB2A*, *AtHB7* and *AtABF3* under normal growth conditions. Phenotype of wild-type and peanut transgenic lines (L1 & L7) co-expressing *AtDREB2A*, *AtHB7* and *AtABF3* (**a**). Net photosynthesis (A), stomatal conductance (gs) and *in-vivo* activity of PSII (Φ_PSII_) of wild type (WT) and transgenic lines (**b**). qRT-PCR showing the relative expression of *AtDREB2A* (2A), *AtHB7* (HB7) and *AtABF3* (ABF3) in selected transgenic (L1 & L7) lines (**c**). RT-PCR showing the expression pattern of target genes in transgenic lines, L1 and L7 (**d**).

Selected transgenic lines that had phenotypes similar to the wild type (Figure 1a) were evaluated under greenhouse conditions. We did not notice any significant difference in a few physiological parameters, such as photosynthetic rate, stomatal conductance and Φ_PSII_ between wild type and transgenic plants under normal conditions (Figure 1b). Quantitative RT-PCR analysis revealed expression of all three transgenes in selected transgenic lines (Figure 1c). *AhHSP70*, *AhSerine threonine kinase* like protein and *AhRing box 1*, the downstream target genes of the transgenes, also showed increased expression in transgenic lines (Figure 1d).

### Evaluation of transgenic lines at seedling stage

**Figure 2:**
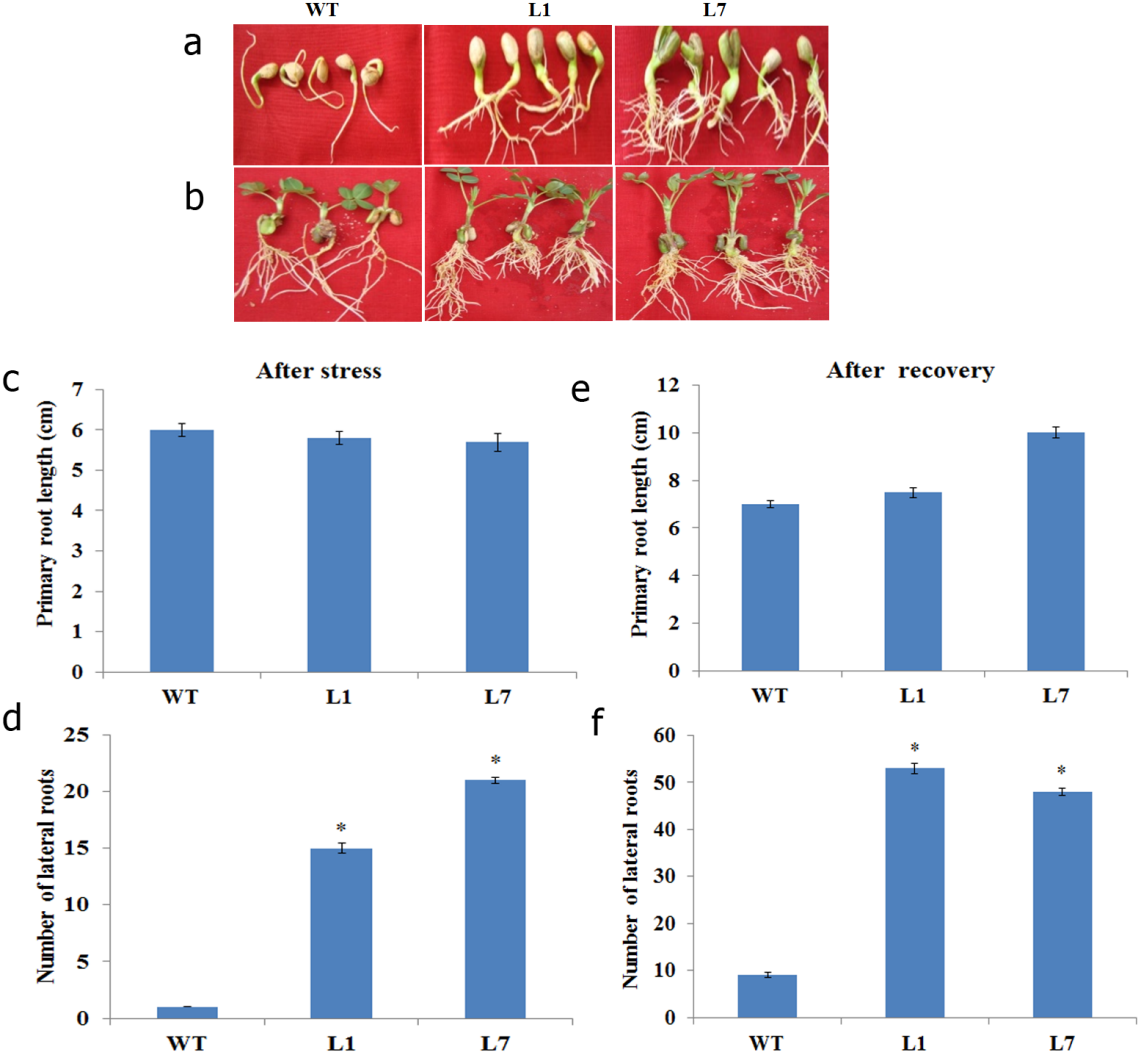
Performance of selected transgenic lines (L1 & L7) co-expressing *AtDREB2A*, *AtHB7* and *AtABF3* under mannitol-induced stress at seedling stage. The seedlings were exposed to mannitol (200 mM) for seven days and photographed (**a**). The seedlings were then allowed to recover from stress for 15 days (**b**). The relative root growth after stress (**c & d**) and recovery (**e & f**) was recorded. The bar represents the mean ± SE (n = 8) (student’s t test; *P<0.05 versus wild-type).

**Figure 3:**
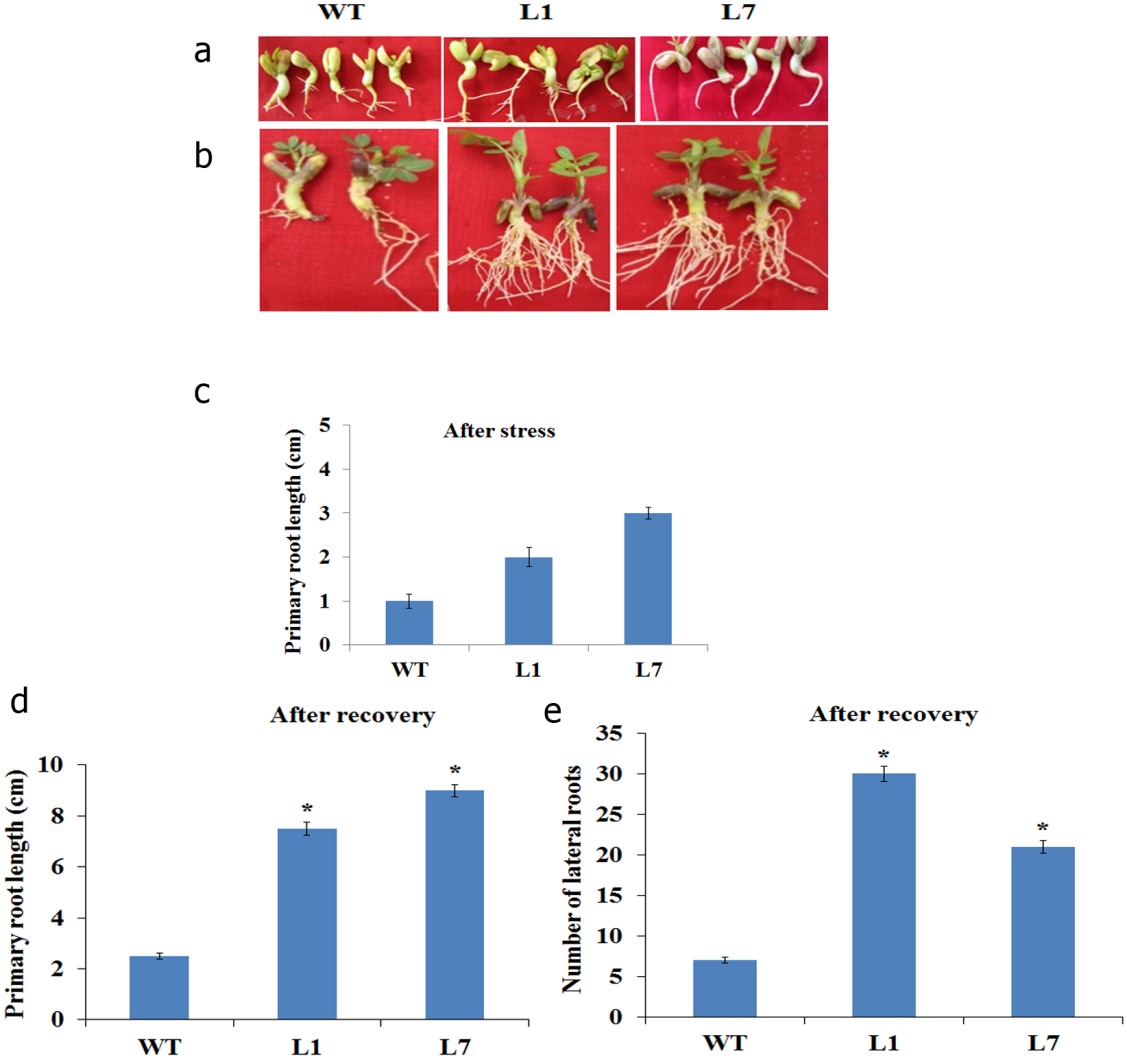
Phenotype of *AtDREB2A*, *AtHB7* and *AtABF3* co-expressing peanut transgenic lines under NaCl-induced stress at seedling stage. The seedlings were exposed to NaCl (200 mM) for seven days and photographed (**a**). The seedlings were then allowed to recover from stress for 15 days (**b**). The relative root growth after stress (**c**) and recovery (**d & e**) were recorded. The bar represents the mean ± SE (n = 8) (student’s t test; *P<0.05 versus wild-type).

Significant difference in the overall growth was noticed between wild type and transgenic seedlings under mannitol- and NaCl-induced stress (Figure 2 and 3, respectively). Although there was no significant difference in primary root length under mannitol stress (Figure 2a and c), there was profuse growth of lateral roots in transgenic lines (Figure 2d). Recovery after stress alleviation was significantly better in transgenic plants as evidenced by increased primary root length (Figure 2b and e) and lateral root numbers in mannitol induced osmotic stress (Figure 2f). Similarly, there was no significant difference in primary root length between wild type and transgenic plants under NaCl-induced stress (Figure 3a and c). After alleviation from NaCl-induced stress (Figure 3b), we noticed a significant increase in primary root length (Figure 3d) and the number of lateral roots (Figure 3e) in transgenic lines compared to wild type.

### Evaluation of transgenic lines at whole plant level

**Figure 4:**
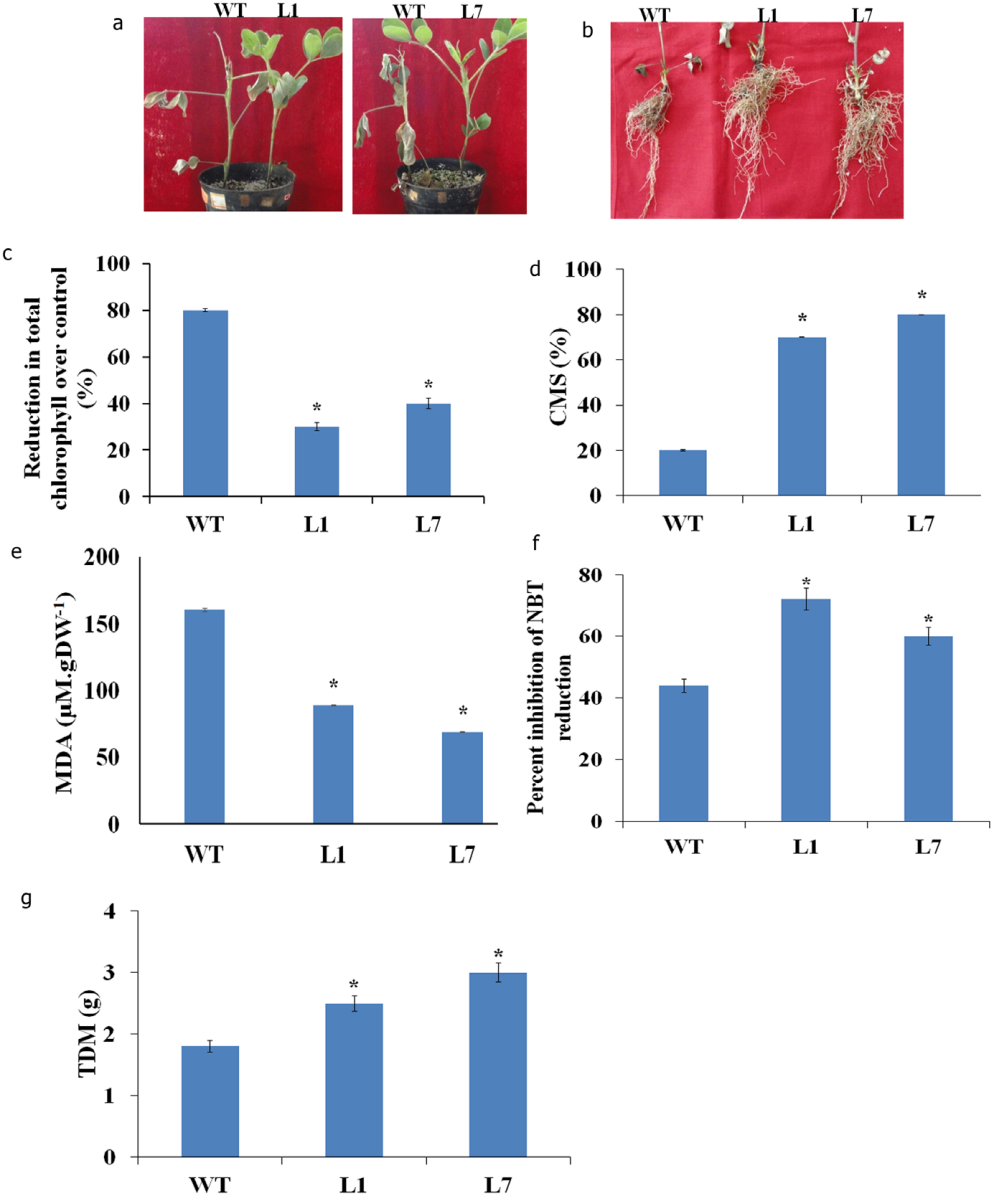
Response of peanut transgenic plants co-expressing *AtDREB2A*, *AtHB7* and *AtABF3* to salinity stress. Salinity stress (250 mM, NaCl) was imposed to three weeks old plants for 10 days. Shoot (**a**) and root (**b**) phenotypes, reduction in chlorophyll content (**c**), cell membrane stability (CMS) (**d**), lipid peroxidation (expressed as MDA content) (**e**), SOD activity (expressed as percent inhibition in NBT reduction) (**f**) and total dry matter (TDM) (**g**) recorded 10 days after stress are presented. The bar represents the mean ± SE of triplicate experiments (student’s t test; *P<0.05 versus wild-type).

The select transgenic lines were tested for the abilities to withstand salinity and drought stress. After exposure to NaCl-induced stress, wild type plants exhibited early symptoms of chlorosis, growth retardation and tip necrosis compared to transgenic plants (Figure 4a and c). Root biomass of the transgenic plants was significantly greater under stress compared to that of wild type (Figure 4b). Cell membrane stability was higher in the transgenic lines (70 and 80% in L1 and L7, respectively) compared to wild type plants (20%, Figure 4d). Under stress the MDA level was significantly more (35%) in wild type compared to transgenic plants (Figure 4e). The activity of SOD, a reactive oxygen species (ROS) scavenging enzyme was significantly (p<0.05) higher in transgenic lines compared to wild type plants (Figure 4f). The biomass of wild type plants (1.6 g DW/plant) was significantly less under stress compared to transgenic plants (3 g DW/plant) (Figure 4g).

**Figure 5:**
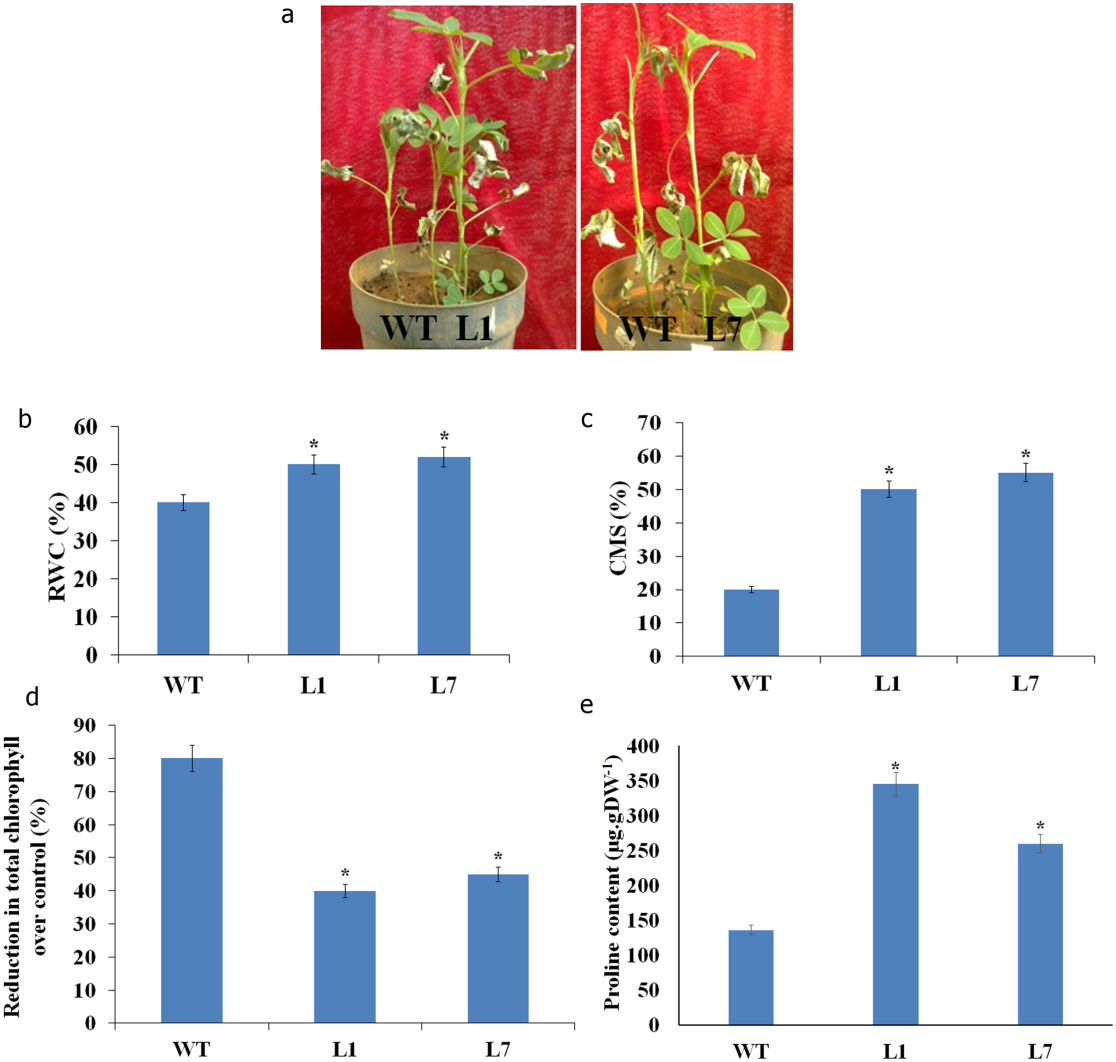
Response of peanut transgenic plants co-expressing *AtDREB2A*, *AtHB7* and *AtABF3* to drought stress at vegetative stage. Plants were gradually exposed to drought stress by controlled irrigation and maintained at 20% field capacity for a week. Phenotype of wild type (WT) and transgenic plants (L1 & L7) recorded after recovery from drought stress (**a**). The relative water content (RWC, **b**), cell membrane stability (CMS, **c**) reduction in chlorophyll content (**d**), and proline content (**e**) were assessed at 30% FC. The bar represents the mean ± SE (student’s t test; *P<0.05 versus wild-type).

Although there was stress induced leaf wilting in both wild type and transgenic lines under severe drought stress, the symptoms appeared much earlier (within seven days after stress imposition) in wild type, with significant phenotypic difference under stress. The transgenic plants regained growth after stress alleviation where as wild type failed to recover (Figure 5a). The relative water content was significantly less in wild type (40%) compared to transgenic lines (50%) under stress (Figure 5b). Stress effect was more pronounced in wild type as evidenced by increased membrane damage (Figure 5c) and reduction in total chlorophyll content (Figure 5d) compared to transgenic lines. Similarly, there was significant increase in proline content in transgenic lines compared to wild type under drought stress (Figure 5e).

### *In-vitro* leaf disc assay

#### Oxidative stress

**Figure 6:**
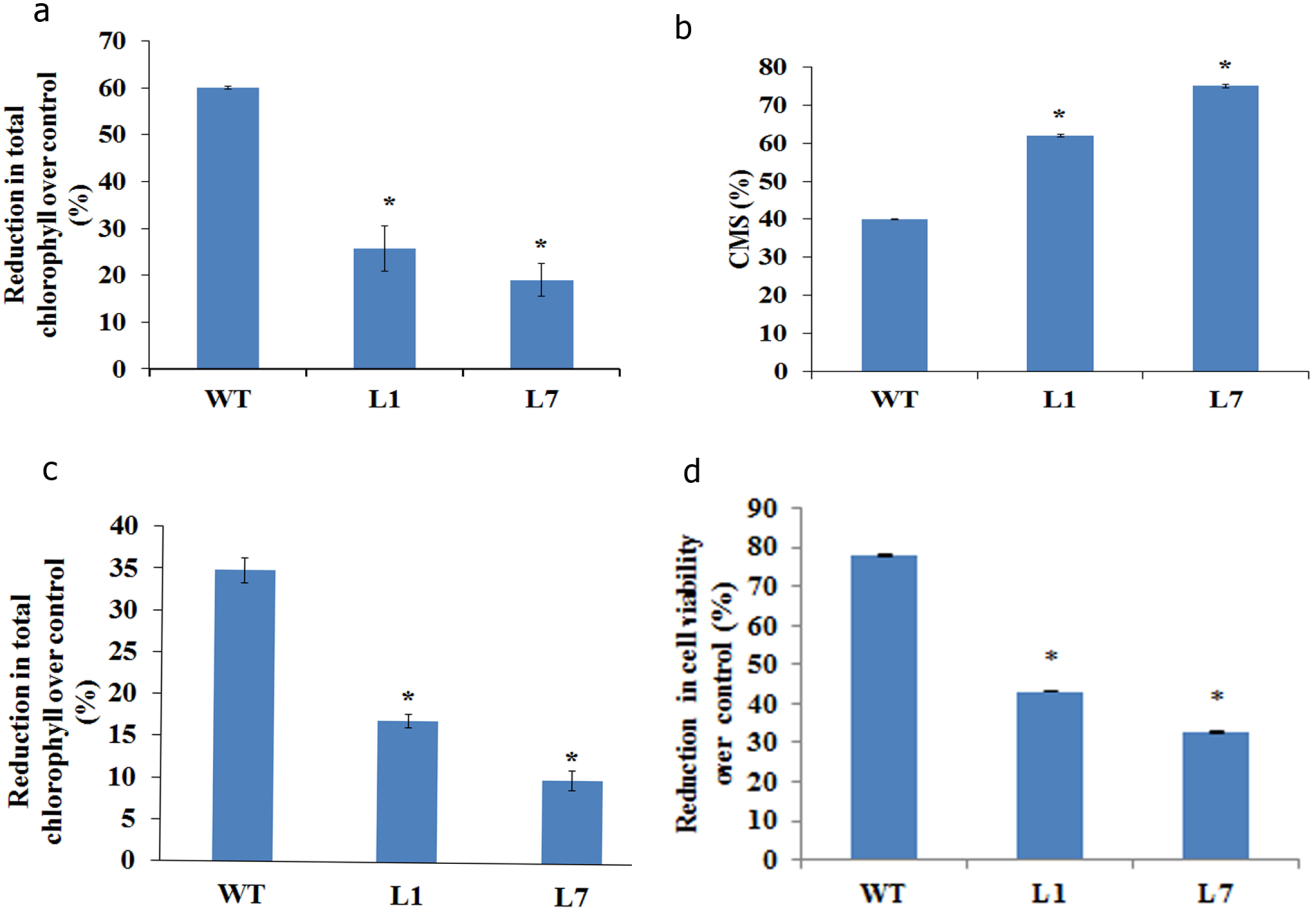
Response of peanut transgenic plants co-expressing *AtDREB2A*, *AtHB7* and *AtABF3* to oxidative stress and ethrel-induced senescence. For oxidative stress, leaf discs were incubated in methyl viologen (5 µM) overnight and exposed to light (1200 µmol.m^−2^.s^−1^) for 1 h. The effect of stress was assessed by estimating reduction in chlorophyll content (**a**) and cell membrane stability (CMS) (**b**). For inducing senescence, leaf discs were incubated in ethrel (1200 ppm) overnight and reduction in chlorophyll content (**c**) and cell viability (**d**) was estimated.

Leaf tissues were treated with methyl viologen to induce oxidative stress. Wild type plants showed 60% reduction in chlorophyll as against 20 and 30% in transgenic lines, L1 and L7 respectively (Figure 6a). Under oxidative stress, there was significant reduction in cell membrane damage in transgenic plants (60 and 75% in L1 and L7 respectively) compared to wild type plants (40%, Figure 6b).

#### Ethrel induced senescence

There was delay in induction of senescence in transgenic lines compared to wild type during ethrel treatment. After 24 h of ethrel treatment, wild type showed 35% reduction in chlorophyll content, which was significantly higher compared to transgenic lines (15 and 10% in L1 and L7 respectively, Figure 6c). There was also significant reduction in cell viability in wild type plants (80%) compared to transgenic lines (Figure 6d).

### Expression analysis of stress responsive target genes

**Figure 7:**
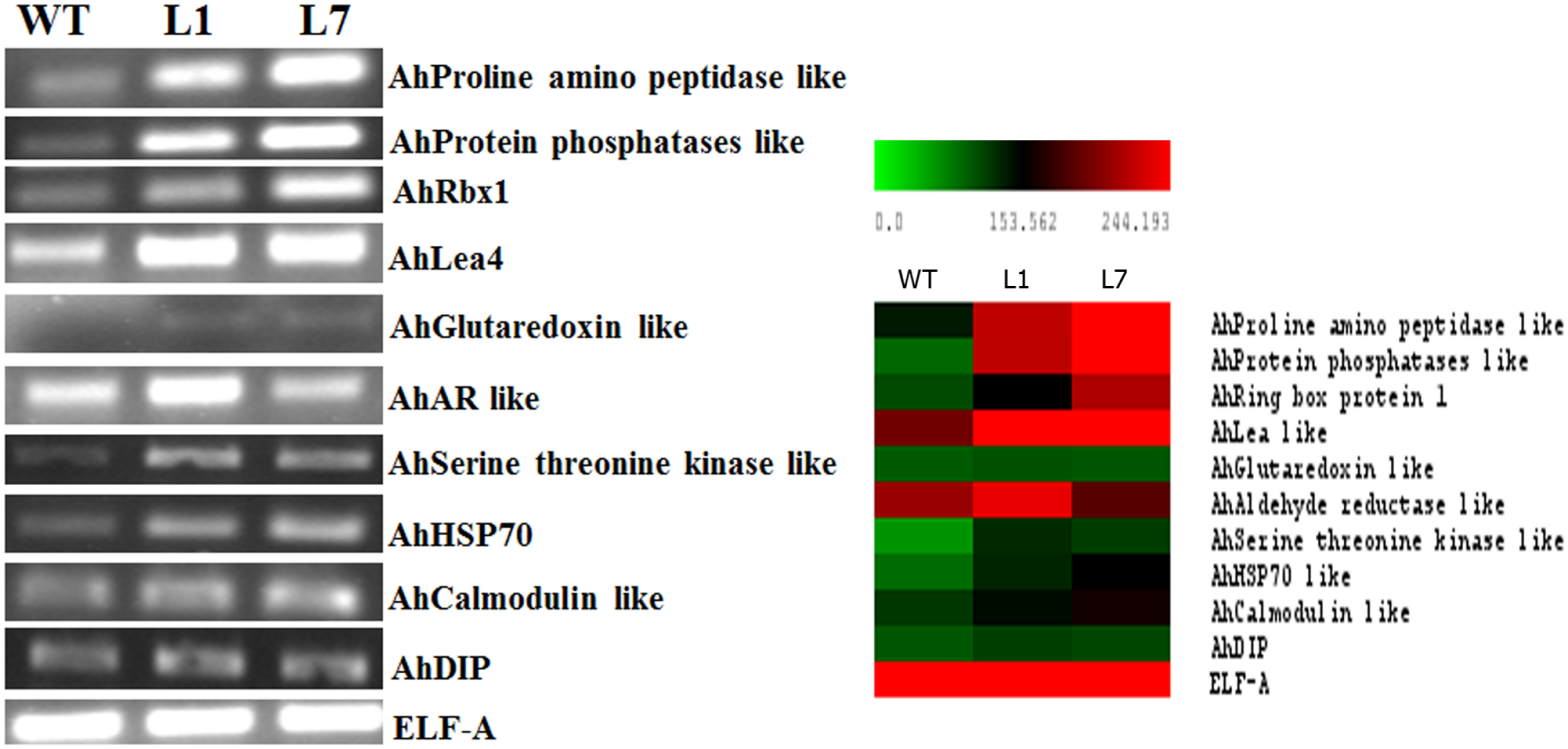
Expression of *AtDREB2A*, *AtHB7* and *AtABF3* target genes in wild type and transgenic plants under drought stress condition. The transcript levels of nine downstream genes were determined by RT-PCR in drought stressed wild type (WT) and transgenic lines (L1 & L7). The, eukaryotic elongation factor (*ELF-A*) was used as internal control. The downstream genes used for expression studies were *AhProline amino peptidase* like protein; *AhRing box protein1* (*AhRbx1*); *Late embryogenesis abundant 4* (*AhLEA4*); *AhGlutaredoxin* like protein; *AhAldehyde reductase* (*AhAR*) like protein; *AhSerine threonine kinase* like protein; *Heat shock Protein70* (*AhHSP70*); *AhCalmodulin* like protein; *Dehydration inducible protein* (*AhDIP*).

To study the expression of a few genes involved in stress tolerance, the downstream target genes of each TF was selected based on the information on TF binding sites using STIF database. Under stress condition, expression of *AhHSP70* was more in transgenic lines compared to wild type. Similarly, expression of *AhAldehyde reductase* (*AhAR*) in transgenic lines was two-fold more than that in wild type. The expression of dehydration inducible protein (*AhDIP*) was apparently enhanced in transgenic lines. *AhLEA4* protein showed higher expression under stress in transgenics compared to the wild type plants. In addition, the expression levels of *AhProline amino peptidase*, *AhGlutaredoxin*, *AhRing box protein1*, *AhSerine threonine kinase* like protein and *AhCalmodulin* like protein were more than wild type in transgenic lines under stress conditions (Figure 7).

## Discussion

In plants, acclimation to abiotic stress tolerance is governed by multiple traits among which cellular tolerance (CT) contributes significantly during all stages of growth and development. Interaction of multiple genes and pathways is required for overall CT under stress. Many processes like osmotic adjustment, cell cycle regulation, protein turnover and removal of toxic compounds including reactive oxygen species (ROS) scavenging, are considered as the components of CT mechanism. The genes that are upregulated or induced under stresses are linked to multiple tolerance pathways, and some of the candidate genes have been well characterised both in model system and crop plants [41], [42]. Amongst the various stress responsive genes, TFs play a crucial role as there are many findings to indicate their relevance in imparting stress tolerance [43]–[45]. The stress responsive TFs could be induced in ABA-independent or ABA-dependent manner and the interaction of the elements of these pathways determine the levels of tolerance. The *ABF/AREB* protein of ABA dependent pathway interacts physically with *DREBs/CBF* of ABA independent pathway [46]. *DREB2A* gene expression under osmotic stress is regulated by *ABRE/ABF* TFs [47]. It is likely that co-expression of specific TFs from both the pathways can enhance the expression of multiple downstream targets required for improved stress tolerance. Stress responsive genes have multiple TF binding sites on their promoters and hence interaction of different TFs seems to be essential for activation of stress responsive downstream genes. From this view, attempts have also been made to develop a strategy to co-express TFs to improve adaptive responses [14], [48], [49]. Co-expression of *AtMYC* and *AtMYB2* leads to enhanced expression of a few downstream genes like *rd22* and *ADH1* [49]. Simultaneous expression of *AtbHLH7* and *AtWRKY28* enhanced the expression of genes having either of the two TF binding sites [14].

We used well characterised TFs regulating ABA-independent (*AtDREB2A*) and ABA-dependent pathways (*AtABF3* and *AtHB7*) for co-expression in peanut to improve CT to abiotic stresses. In earlier studies, the relevance of these TFs has been demonstrated independently through constitutive expression. The *DREB2A* belongs to Apetala2/Ethylene-responsive element binding factor (*AP2/ERF*) family which can impart tolerance to salinity and drought stresses [50]–[52]. Overexpression of *DREB2A* induced more than 303 stress responsive downstream genes [53]. The other TF, *AtHB7* used here is an ABA- and drought-inducible gene [54]. Overexpression and ectopic expression of *AtHB7* in *Arabidopsis* and tomato, respectively, resulted in enhanced dehydration tolerance [55], [56]. The analysis of mutant and transgenic plants in *Arabidopsis* indicated that *AtHB7* expression depends on plant developmental stage and the environmental conditions [57]. *AtHB7* plays a major role in plant growth and development, which increases the chlorophyll content and delays senescence in the later stage of plant development [57]. The third protein expressed is a basic leucine zipper (bZIP) family member, an *ABRE* binding factor (*ABF3*) that has been functionally characterized as a component of ABA signalling [58]. *ABF3* is induced by ABA, salt, cold or drought stress [59]–[61]. Over-expression of *ABF3* in *Arabidopsis* enhanced tolerance to drought [62]. Ectopic expression of *AtABF3* in lettuce also enhanced tolerance under drought and cold stress [63]. Since the regulatory genes used can activate multiple stress-tolerance pathways, co-expression resulted in improved tolerance to different abiotic stresses.

We noticed significant improvement in salinity tolerance in transgenic plants, although the content of Na^+^ and K^+^ was similar between wild type and transgenic lines (Table S2 in File S1). Therefore, difference in uptake of ions has not contributed for the response noticed in the transgenic lines and the transgenes expression might have imparted tolerance by modifying stress related gene expression. It is certain that management of secondary stress induced by ROS is very important and stress adapted plants have an efficient mechanism to scavenge ROS [64]. The gene *ABF* belonging to bZIP family has been shown to be associated with oxidative stress management. The transgenic lines showed increased activity of scavenging enzyme, SOD compared to wild type plants suggesting the role of *AtABF3* in activating ROS scavenging machinery. An important physiological mechanism associated with tolerance to water deficit condition is osmotic adjustment [65] and proline, a compatible osmolyte synthesised in plants plays an important role in osmotic adjustment [66]. We noticed significant increase in proline content in transgenic lines compared to wild type plants, indicating efficient activation of osmotic adjustment mechanisms required for CT under drought. The transgenic lines showed delayed senescence under ethylene-induced stress, which could be due to the expression of *AtHB7,* the TF having role in delaying senescence as demonstrated earlier [57]. In previous study, although there was reduction in stomatal conductance in transgenic tomato plant expressing *AtHB7* [56], we did not notice such phenotype in our transgenic plants. This could be due to interactive effect of co-expression of TFs belonging to ABA-dependent and ABA-independent pathways. Additionally, there seems to be multiple regulatory factors and elements involved in coordinated expression of *AtHB7* [57], which needs to be examined. Significant improvement in abiotic stress tolerance in the co-expressing lines suggests that targeted trait manipulation is possible by this type of approaches.

To examine possible downstream target genes of the three TFs, promoters of some stress responsive genes were analysed by using STIF database (Stress Gene Transcription Factor). Over 800, 1558 and 154 downstream target genes were found to have *DRE*, *ABRE* and *HDE* cis elements, respectively in their promoter regions (data not shown). Therefore, it is likely that simultaneous expression of these three TFs would have activated multiple target gene expression leading to improved stress tolerance in transgenic lines.

We analysed the expression of a few downstream genes associated with CT in two promising transgenic lines. The upstream regulatory genes encoding *Serine threonine kinase* like (At2G25880) and *Calmodulin* (At5G37780) like proteins, which contain *DRE* and *ABRE* cis elements in their promoter region [67], [68], were activated in transgenic lines. Stress responsive genes, *Lea4* (AT2G21490; having *HDE*, *ABRE* and *DRE*), *Ring box 1 protein* (*Rbx1*; At3G42830, having *HDE*) and *HSP70* (AT3G12580, having *DRE*) were up-regulated in transgenic lines compared to wild type plants. The *Lea* (*NtERD10B*) was reported earlier to be up-regulated in transgenic tobacco plants expressing *PgDREB2A* [69], which stabilizes and maintains the protein structure under stress. The *HSP70*, which act as molecular chaperones [70], was up-regulated in transgenic lines. Similarly, *Ring box 1* (*Rbx1*) protein, a highly stress responsive protein involved in protein degradation under stress by ubiquitin proteosome pathway [71], was also up-regulated in the transgenic plants under stress. Up-regulation of *Lea* and *HSP70* would have improved protein stability, and *Rbx1* would have contributed for efficient degradation of unfolded or misfolded proteins under stress in transgenic peanut plants.

It is likely that increased expression of detoxifying enzymes like *AhAldehyde reductase* (At5G01670, having *DRE*, *ABRE* and *HDE*) and *AhGlutaredoxin* (AT3G15660, having *HDE*), in transgenic lines contributed for efficient management of oxidative stress which resulted in reduced lipid peroxidation. Expression of *AhProline amino peptidase* (At4G30910; having *ABRE*) and *Drought Induced Protein* (*DIP*; At4G15910 with *ABRE* and *DRE*), was found to be more in transgenic plants than wild type indicating that the TFs overexpressed activated wide range of target genes.

In summary, our study suggests that simultaneous expression of multiple TFs under stress conditions is useful in activating diverse events associated with CT. This is quite plausible considering the fact that stress response of plants is often a net-worked event at different hierarchies, from receipt of, to response to signals [72], [73]. From the current study, however, it is not clear if the net response of plants as measured by their CT is due to additive or synergistic interactions among the three TFs in the transgenic plants. Ideally, it would have been appropriate to compare the effect of the three TFs with that of single TF. However due to the relatively high degree of recalcitrant nature of peanut to transformation [74], [75], this was not attempted in this study. But by extrapolation of the single gene effects studied in other plant systems [7], [23], [24], it appears that in the triple gene transgenic plants there could be interactive effects, which together increases the CT under stress. In fact, some studies have shown the possibility of additive effect on co-expression of multiple genes [14], [48], [49]. These studies, as do our results, suggest a promising approach of using multiple genes to harness the interactive/additive effects towards improving abiotic stress tolerance of plants. The stacking of relevant genes that are critically involved in contributing to CT can optimise plants’ adaptation to abiotic stresses.

## Supporting Information

File S1 **Figure S1. Generation of peanut (** ***Arachis hypogaea*** **L.,** ***cv*** **. TMV2) transgenic plants co-expressing three transcription factors (TFs).** Vector map representing the T-DNA region of the plant expression vector (*pKM12GW-AtDREB2A-AtHB7-AtABF3*) (**a**). Different stages of peanut transformation (**b–g**). Germination of sterilized peanut seeds on sterilized wet filter paper bridge for obtaining explants (**b**). Selection of putative transgenic plants on SIM with kanamycin (125 mg/L) (**c**). Completely green multiple shoot on SPM three weeks after transformation on kanamycin selection media (125 mg/L) (**d**). Elongation of putative transgenic plants on SEM with GA (1 mg/L) (**e**). Profusely rooted putative transgenic plants on RIM (**f**). Acclimation of hardened transgenic plants to greenhouse conditions (**g**). Selection of T_1_ transgenic plants on sand containing ½ MS media (**h**). **Figure S2. Sequence of** ***AtDREB2A*** **,** ***AtHB7*** **and** ***AtABF3*** **amplified from genomic DNA of transgenic peanut plants.** The integration of transgenes was confirmed by PCR using genomic DNA of transgenic peanut plants as template. The amplified product was confirmed by sequencing. The sequence of *AtDREB2A* (**i**), *AtHB7* (**ii**) and *AtABF3* (**iii**) are presented. **Table S1. List of primers used for vector construction, integration and expression analysis of transgenes, and a few stress responsive target genes in peanut. Table S2. Na^+^ and K^+^ content in wild type and transgenic plants (L1 & L7) under control and salinity stress.** The data represents the mean ± SD (n = 5) (student’s t test; *P<0.05 versus wild-type).
(ZIP)
