# Supplementary figures and images for: Simultaneous Expression of Abiotic Stress Responsive Transcription Factors, AtDREB2A, AtHB7 and AtABF3 Improves Salinity and Drought Tolerance in Peanut (Arachis hypogaea L.)
Source: PLoS One. 2014 Dec 4;9(12):e111152. doi: 10.1371/journal.pone.0111152 (PMC4256372; doi:10.1371/journal.pone.0111152)

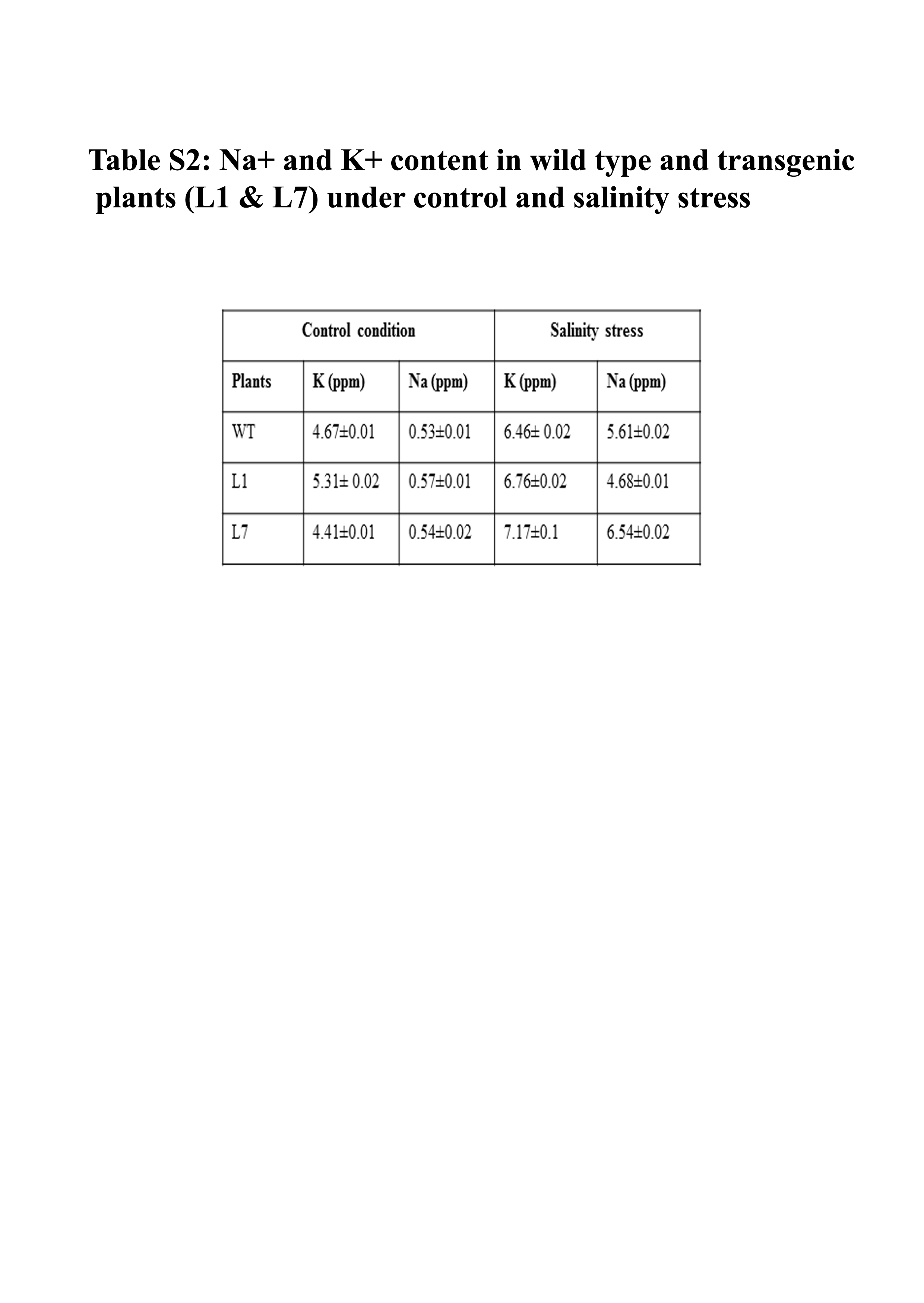

Supplement: File S1 — Figure S1. Generation of peanut ( Arachis hypogaea L., cv . TMV2) transgenic plants co-expressing three transcription factors (TFs). Vector map representing the T-DNA region of the plant expression vector (pKM12GW-AtDREB2A-AtHB7-AtABF3) (a). Different stages of peanut transformation (b–g). Germination of sterilized peanut seeds on sterilized wet filter paper bridge for obtaining explants (b). Selection of putative transgenic plants on SIM with kanamycin (125 mg/L) (c). Completely green multiple shoot on SPM three weeks after transformation on kanamycin selection media (125 mg/L) (d). Elongation of putative transgenic plants on SEM with GA (1 mg/L) (e). Profusely rooted putative transgenic plants on RIM (f). Acclimation of hardened transgenic plants to greenhouse conditions (g). Selection of T1 transgenic plants on sand containing ½ MS media (h). Figure S2. Sequence of AtDREB2A , AtHB7 and AtABF3 amplified from genomic DNA of transgenic peanut plants. The integration of transgenes was confirmed by PCR using genomic DNA of transgenic peanut plants as template. The amplified product was confirmed by sequencing. The sequence of AtDREB2A (i), AtHB7 (ii) and AtABF3 (iii) are presented. Table S1. List of primers used for vector construction, integration and expression analysis of transgenes, and a few stress responsive target genes in peanut. Table S2. Na+ and K+ content in wild type and transgenic plants (L1 & L7) under control and salinity stress. The data represents the mean ± SD (n = 5) (student’s t test; *P<0.05 versus wild-type). (ZIP) [file pone.0111152.s001.zip › Table S2.tif]

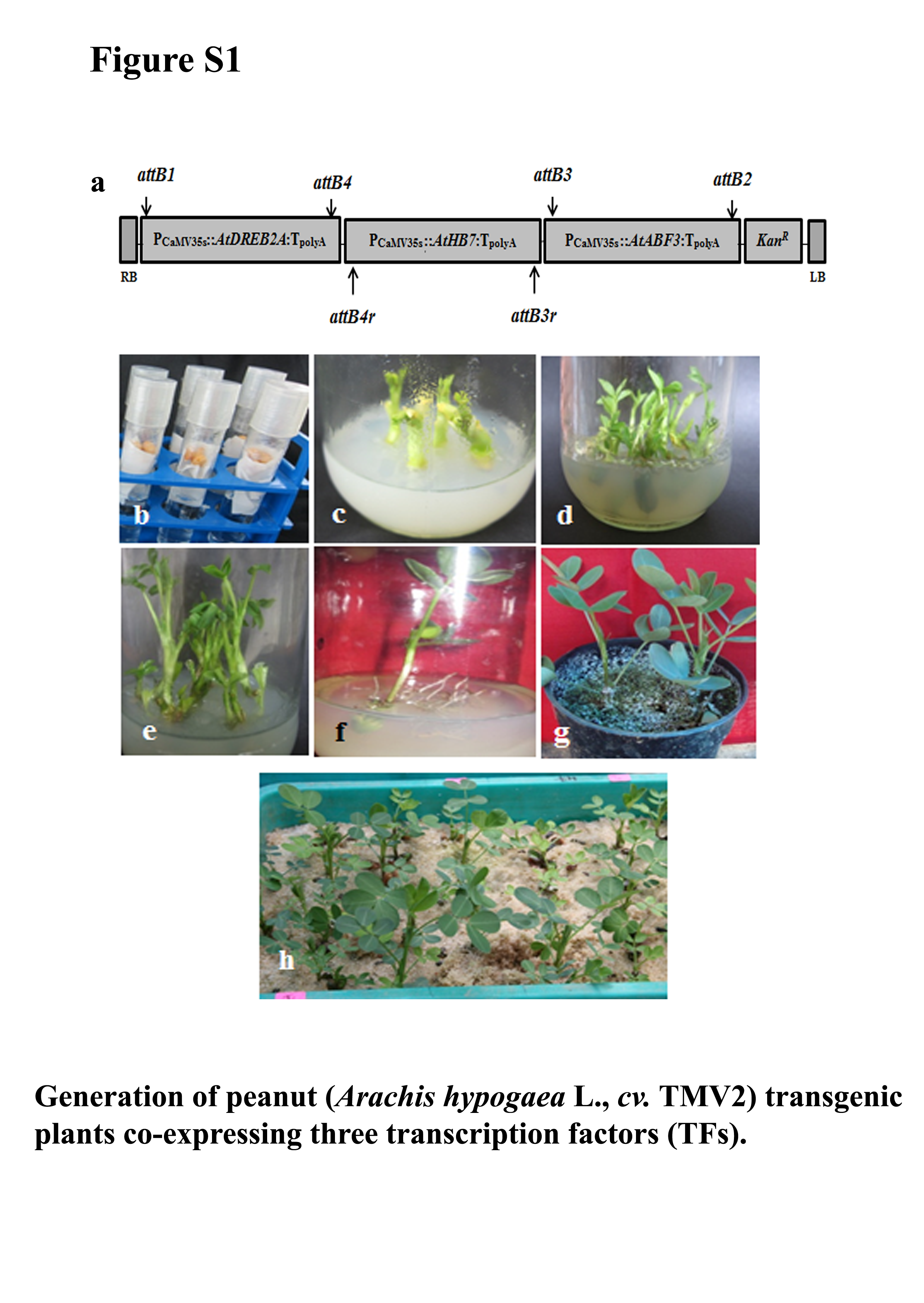

Supplement: File S1 — Figure S1. Generation of peanut ( Arachis hypogaea L., cv . TMV2) transgenic plants co-expressing three transcription factors (TFs). Vector map representing the T-DNA region of the plant expression vector (pKM12GW-AtDREB2A-AtHB7-AtABF3) (a). Different stages of peanut transformation (b–g). Germination of sterilized peanut seeds on sterilized wet filter paper bridge for obtaining explants (b). Selection of putative transgenic plants on SIM with kanamycin (125 mg/L) (c). Completely green multiple shoot on SPM three weeks after transformation on kanamycin selection media (125 mg/L) (d). Elongation of putative transgenic plants on SEM with GA (1 mg/L) (e). Profusely rooted putative transgenic plants on RIM (f). Acclimation of hardened transgenic plants to greenhouse conditions (g). Selection of T1 transgenic plants on sand containing ½ MS media (h). Figure S2. Sequence of AtDREB2A , AtHB7 and AtABF3 amplified from genomic DNA of transgenic peanut plants. The integration of transgenes was confirmed by PCR using genomic DNA of transgenic peanut plants as template. The amplified product was confirmed by sequencing. The sequence of AtDREB2A (i), AtHB7 (ii) and AtABF3 (iii) are presented. Table S1. List of primers used for vector construction, integration and expression analysis of transgenes, and a few stress responsive target genes in peanut. Table S2. Na+ and K+ content in wild type and transgenic plants (L1 & L7) under control and salinity stress. The data represents the mean ± SD (n = 5) (student’s t test; *P<0.05 versus wild-type). (ZIP) [file pone.0111152.s001.zip › Figure S1.tif]

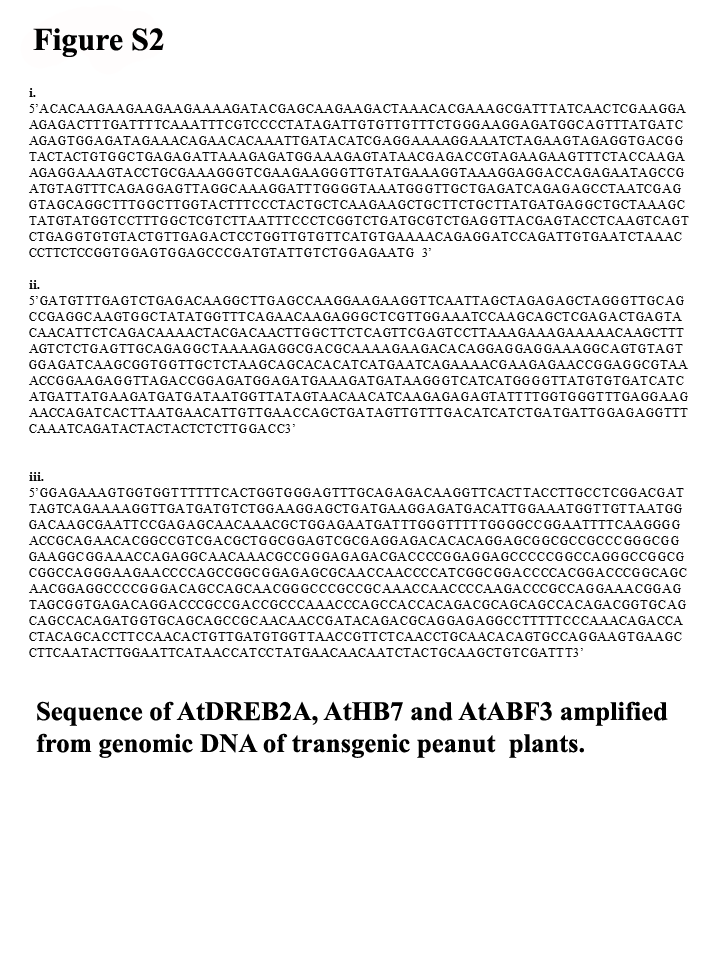

Supplement: File S1 — Figure S1. Generation of peanut ( Arachis hypogaea L., cv . TMV2) transgenic plants co-expressing three transcription factors (TFs). Vector map representing the T-DNA region of the plant expression vector (pKM12GW-AtDREB2A-AtHB7-AtABF3) (a). Different stages of peanut transformation (b–g). Germination of sterilized peanut seeds on sterilized wet filter paper bridge for obtaining explants (b). Selection of putative transgenic plants on SIM with kanamycin (125 mg/L) (c). Completely green multiple shoot on SPM three weeks after transformation on kanamycin selection media (125 mg/L) (d). Elongation of putative transgenic plants on SEM with GA (1 mg/L) (e). Profusely rooted putative transgenic plants on RIM (f). Acclimation of hardened transgenic plants to greenhouse conditions (g). Selection of T1 transgenic plants on sand containing ½ MS media (h). Figure S2. Sequence of AtDREB2A , AtHB7 and AtABF3 amplified from genomic DNA of transgenic peanut plants. The integration of transgenes was confirmed by PCR using genomic DNA of transgenic peanut plants as template. The amplified product was confirmed by sequencing. The sequence of AtDREB2A (i), AtHB7 (ii) and AtABF3 (iii) are presented. Table S1. List of primers used for vector construction, integration and expression analysis of transgenes, and a few stress responsive target genes in peanut. Table S2. Na+ and K+ content in wild type and transgenic plants (L1 & L7) under control and salinity stress. The data represents the mean ± SD (n = 5) (student’s t test; *P<0.05 versus wild-type). (ZIP) [file pone.0111152.s001.zip › Figure S2.tif]

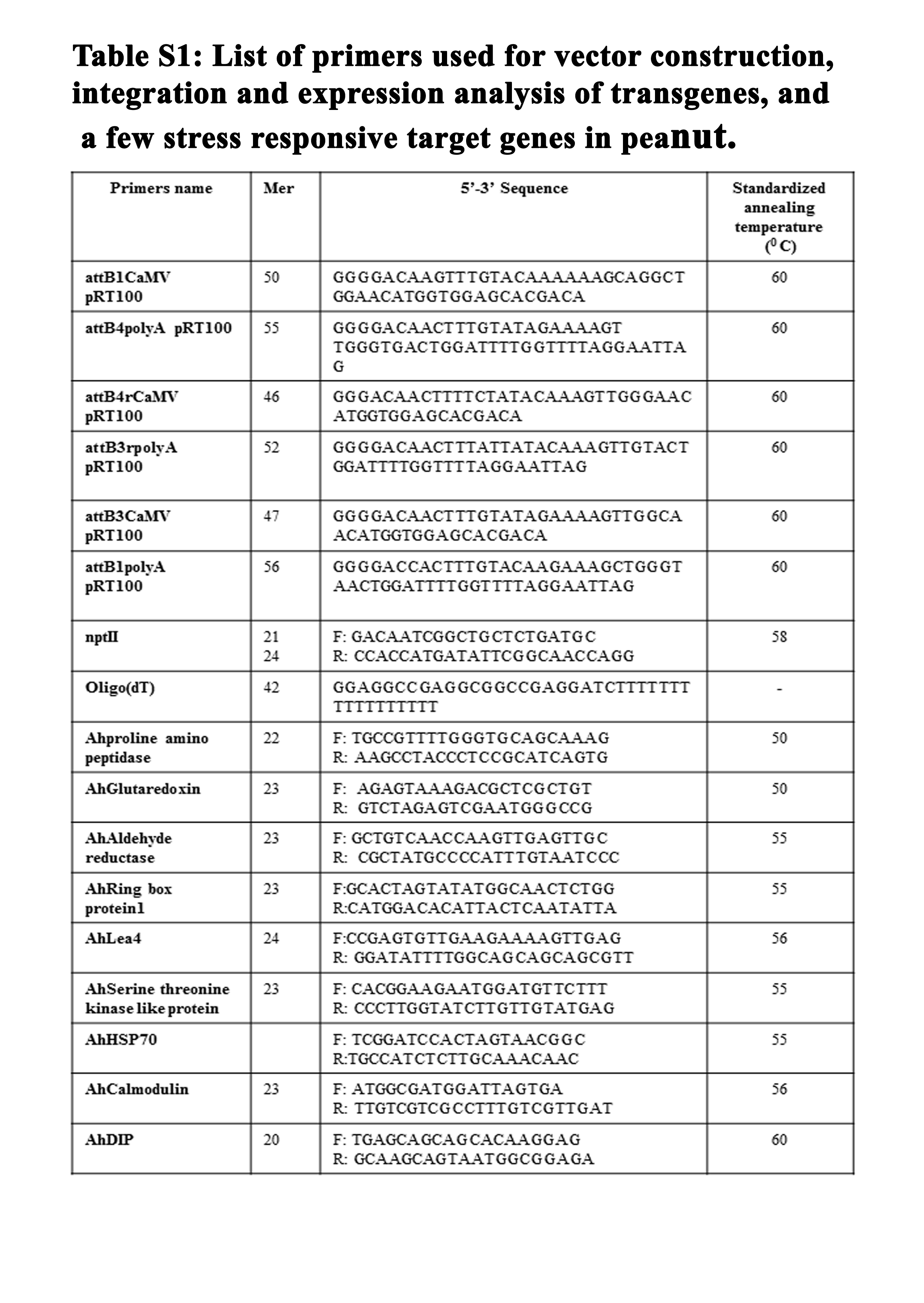

Supplement: File S1 — Figure S1. Generation of peanut ( Arachis hypogaea L., cv . TMV2) transgenic plants co-expressing three transcription factors (TFs). Vector map representing the T-DNA region of the plant expression vector (pKM12GW-AtDREB2A-AtHB7-AtABF3) (a). Different stages of peanut transformation (b–g). Germination of sterilized peanut seeds on sterilized wet filter paper bridge for obtaining explants (b). Selection of putative transgenic plants on SIM with kanamycin (125 mg/L) (c). Completely green multiple shoot on SPM three weeks after transformation on kanamycin selection media (125 mg/L) (d). Elongation of putative transgenic plants on SEM with GA (1 mg/L) (e). Profusely rooted putative transgenic plants on RIM (f). Acclimation of hardened transgenic plants to greenhouse conditions (g). Selection of T1 transgenic plants on sand containing ½ MS media (h). Figure S2. Sequence of AtDREB2A , AtHB7 and AtABF3 amplified from genomic DNA of transgenic peanut plants. The integration of transgenes was confirmed by PCR using genomic DNA of transgenic peanut plants as template. The amplified product was confirmed by sequencing. The sequence of AtDREB2A (i), AtHB7 (ii) and AtABF3 (iii) are presented. Table S1. List of primers used for vector construction, integration and expression analysis of transgenes, and a few stress responsive target genes in peanut. Table S2. Na+ and K+ content in wild type and transgenic plants (L1 & L7) under control and salinity stress. The data represents the mean ± SD (n = 5) (student’s t test; *P<0.05 versus wild-type). (ZIP) [file pone.0111152.s001.zip › Table S1.tif]
